# *Plagioscion squamosissimus* (Sciaenidae) and *Parachromis managuensis* (Cichlidae): A Threat to Native Fishes of the Doce River in Minas Gerais, Brazil

**DOI:** 10.1371/journal.pone.0039138

**Published:** 2012-06-13

**Authors:** Lucas C. Barros, Udson Santos, José C. Zanuncio, Jorge A. Dergam

**Affiliations:** 1 Departamento de Biologia Animal, Universidade Federal de Viçosa, Viçosa, Minas Gerais State, Brazil; 2 Departamento de Biodiversidade, Evolução e Meio Ambiente, Universidade Federal de Ouro Preto, Ouro Preto, Minas Gerais State, Brazil; Biodiversity Insitute of Ontario – University of Guelph, Canada

## Abstract

The middle section of the lake basin of the Doce River in Minas Gerais State, Brazil is plagued by grave environmental problems, including the introduction of non-native fish, which reduces the biodiversity of this region. This study reports the presence of two newly-detected non-native species in the Doce River Basin. Sampling efforts included gill nets with mesh size of 3 to 12 mm (measured diagonally) and trawling nets, both of which were used in independent field campaigns in 2002 and 2011. The two new invasive Perciform fishes, *Plagioscion squamosissimus* (Heckel 1840) and *Parachromis managuensis* (Günther 1867) were collected in Caratinga and Rio Doce municipalities. These records and other reports on non-native fishes suggest favorable environmental conditions for the establishment of invasive species in this drainage. These invasive species have behavior and diet observed in other wide distribution exotic fish of Rio Doce Basin representing a threat to the 77 native fishes of this region, 37 of which are endangered.

## Introduction

Species introduction in aquatic ecosystems has been documented worldwide [1], with biological invasions threatening native biodiversity and ecosystem function [2]. Non-native species have direct and indirect effects on the structure and functioning of aquatic and terrestrial ecosystems and may cause economic problems, sometimes affecting food supply and human health. Biological invasions also cause more subtle environmental problems such as the introduction of infectious agents and pollution by sex pheromones [3]; therefore controlling non-native species may be very expensive [4] or almost impossible.

Invasions by alien species have increased in recent decades due to trade and tourism between countries and continents. Preventing introductions is the most efficient and economic strategy to address this problem [5]. However, modern transport systems and advances in aquaculture increase the dispersal of animals and plants over biogeographical barriers that would normally hinder them [6]. A low percentage of highly tolerant species become invasive in populations dispersed over large areas [7], [8] and little is known about their actual impact on native fauna [9].

The establishment of non-native species is the third major cause of extinction in fishes throughout the world [10], after habitat fragmentation [11] and habitat alteration [12]. The Red List of Threatened Species in South America reports that non-native species had negative effects on 29% of continental fishes and 30% of amphibian species [13]. The largest river basins in the world and the greatest number of fish species of the Neotropical region are located in Brazil [14] and are threatened by introductions of exotic fish species [15].

The Doce River watershed drains 12% of the territory of Minas Gerais State, Brazil. It contains approximately 150 lakes formed by glacial and interglacial events, and these lakes harbor one-third of the ichthyofauna of this basin [16]. The great biological diversity and geological peculiarities make this lake system very important in conserving the Atlantic Forest Biome [17]. Among the 77 fish species recorded in the Doce River watershed, 37 are of restricted distribution and are thus a priori threatened with extinction [18] due to anthropogenic changes [19] and introduction of exotic fishes [20], [21], the latter being the main factor leading to extinction of native fishes.

This paper reports the occurrence of two new non-native fish species in the upper and middle Doce River in Minas Gerais State, Brazil, which were collected in separate field work activities as part of a long-term monitoring program designed to detect fish introductions in the region.

## Methods

**Figure 1:**
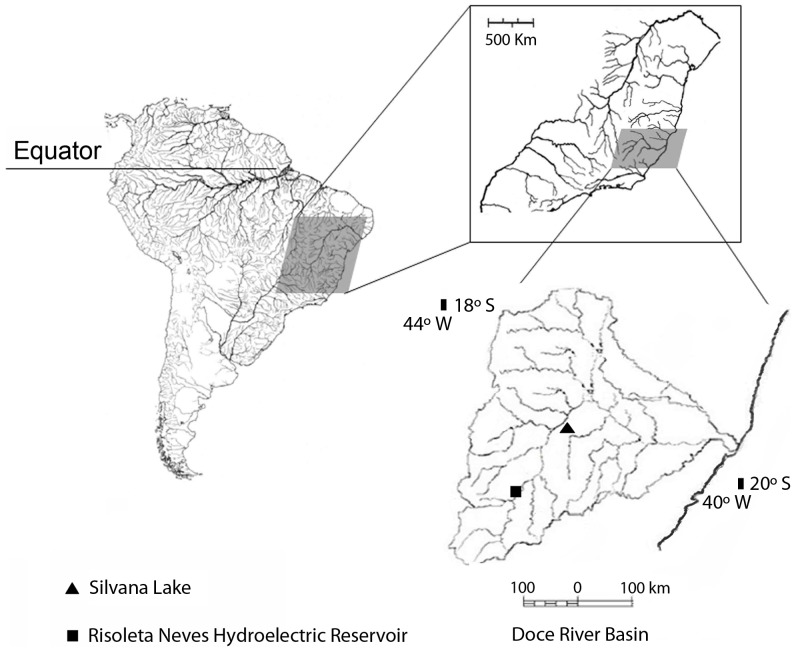
Collection sites in Doce River Basin for *Plagioscion squamosissimus* (triangle) and *Parachromis managuensis* (square).

Field campaigns were conducted in 2002 and 2011 in Silvana Lake in the municipalities of Caratinga and Rio Doce in southeast Minas Gerais State, Brazil, in an area that was originally part of the Atlantic Forest Biome (Fig. 1). Sampling efforts in Silvana Lake were part of the species monitoring program funded by Empresa de Celulose Nipo Brasileira (CENIBRA). In the upper Rio Doce, the activities were included in the ichthyofauna monitoring program in the Risoleta Neves hydroelectric reservoir. Samples were collected using trawling nets (mesh size of 5 mm measured diagonally), and gill nets in the municipality of Caratinga (19°29′34.81″ S, 42°25′28.40 W″) in December 2002 and in the municipality of Rio Doce (20°14′45.14″ S, 42°53′43.58″ W) in June and December 2011. Sampling was performed with mesh nets set at dusk (5:00 p.m.) and retrieved at dawn (6 a.m.). Each set of gill nets (mesh sizes of 3, 4, 5, 7, 8, 10 and 12 mm) were placed at sampling points 1500 meters apart. The distance among gill nets in the same area was 10 meters. Two points were sampled at Silvana Lake and five at the Risoleta Neves hydroelectric reservoir. In each lake, three points1,500 meters apart and amenable to the use of trawl net were sampled with three passages. The hauls were performed between 3:00 and 5:00 p.m.. Animals collected were fixed in 10% formalin, stored in 70% alcohol, and deposited in the collection of the John Moojen Museum of Zoology of the Federal University of Viçosa (MZUFV). Species captured were anaesthetized using clove oil, identified using standard protocols and identification keys of species. Collecting permit SISBIO14975-1 was issued to Prof. Jorge Dergam by the National Institute Chico Mendes of Biodiversity (ICMBio), an authorization valid for collection of all fish taxa occurring in Brazil.

**Figure 2:**
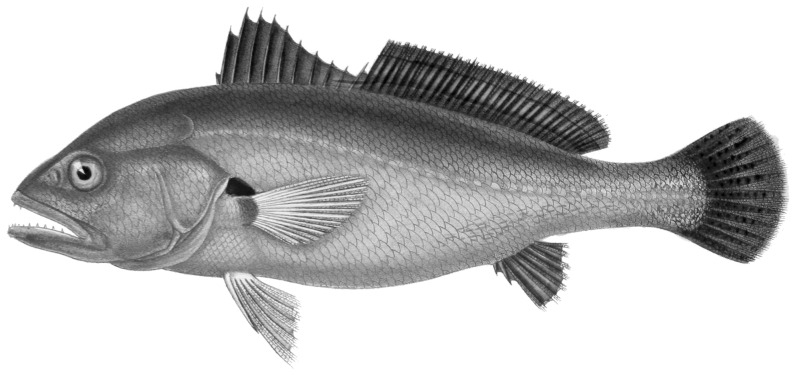
*Plagioscion squamosissimus*, endemic to the Amazonian Basin, collected in Lake Silvana, Municipality of Caratinga, Minas Gerais State, Brazil. Figure modified from Froese and Pauly [37].

**Figure 3:**
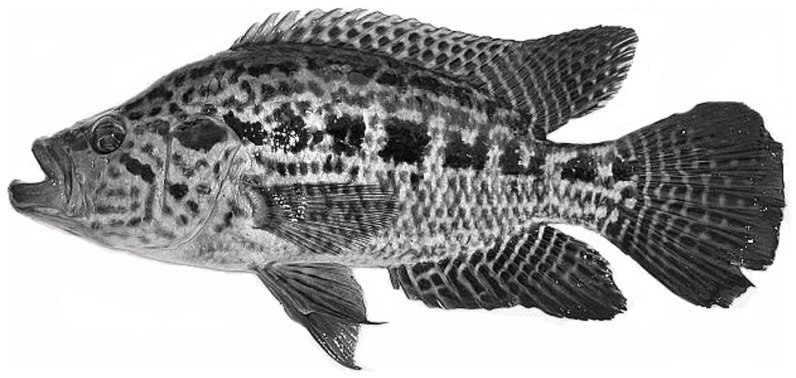
*Parachromis managuensis*, endemic to Central America, collected in the Doce River, Municipality of Rio Doce, Minas Gerais State, Brazil. Figure modified from Froese and Pauly [37].

**Table 1:** Non-native fish species have been captured in the middle Doce River Basin, Minas Gerais, Brazil.

Species		Collectors				
	Godinho et al (1994)	Latini et al (2004)	Vieira (2006)	Alves et al (2007)	Pinto-Coelho (2008)	This study
*Plagioscion squamosissimus*(Heckel, 1840)						X
*Parachromis managuensis*(Günther, 1867)						X
*Cichla monoculus*Spix & Agassiz, 1831	X	X			X	X
*Cicha ocellatus*Bloch & Schneiderm 1801				X		
*Pygocentrus nattereri*Kner, 1858	X	X	X	X	X	
*Astronotus ocellatus*(Agassiz, 1831)		X		X	X	
*Clarias gariepinus*(Burchell, 1882)		X	X	X		
*Oreochromis niloticus*(Linnaeus, 1758)		X	X			X
*Hoplosternum littorale*(Hancock, 1828)		X	X	X	X	X
*Colossoma macropomum*(Cuvier, 1818)		X		X		
*Leporinus macrocephalus*Garavello & Britski, 1998			X	X		
*Piaractus mesopotamicus*(Holmberg, 1887)			X			
*Salminus brasiliensis*(Cuvier, 1816)			X	X		
*Prochilodus costatus*(Cuvier & Valenciennes, 1849)			X	X		
*Prochilodus argenteus*Agassiz, 1829						
*Poecilia reticulata*Peters, 1859			X			X
*Tilapia rendalli*(Boulenger, 1897)			X	X		
*Pogonopoma wertheimeri*(Steindachner, 1867)			X			
*Lophiosilurus alexandri*(Steindachner 1876)			X			
*Pimelodus maculatus*(Lacepède, 1803)			X			
*Aristichthys nobilis*(Richardson, 1845)				X		
*Pseudoplatystoma* sp.			X			
*Pseudoplatystoma* hibrid^1^				X		
*Tambacu* hibrid^2^				X		
*Xiphophorus hellerii*Heckel, 1848				X		
*Ctenopharyngodor idella*(Valenciennes, 1844)				X		
*Cyprinus carpio*Linnaeus, 1758				X		
*Hoplias intermedius*Oyakawa & Mattox, 2009				X		
*Hyphessobrycon eques*(Steindachner, 1882)				X		
*Hypophthalmichthys molitrix*(Valenciennes, 1844)				X		
*Ictalurus punctatus*(Rafinesque, 1818)				X		
*Lepomis gibbosus*(Linnaeus, 1758)				X		
*Metynnis maculatus*Kner, 1858				X		
*Micropterus salmoides*(Lacepède, 1802)				X		
*Piaractus mesopotamicus*Holmberg, 1887				X		

1 Hybrid of *Pseudoplatystoma corruscans* (Spix & Agassiz, 1829) with *Pseudoplatystoma fasciatum* (Linnaeus, 1766).

2 Hybrid of *Colossoma macropomum* with *Piaractus mesopotamicus*.

## Results

Two new non-native Perciform species were recorded for the middle Doce River Valley in Minas Gerais, Brazil. Two specimens of *Plagioscion squamosissimus* (Heckel, 1840), with 20 and 15 cm in length (MZUFV3268) (Fig. 2), were collected in 2002 at Lake Silvana in the municipality of Caratinga. Two specimens of *Parachromis managuensis*, 13 and 10 cm in length (Günther, 1867) (MZUFV3945) (Fig. 3), were found in July and December of 2011 at the upper Doce River in Rio Doce municipality. Other exotic species fish already recorded in the Doce River watershed were also sampled: *Cichla monoculus* Spix & Agassiz, 1831 and *Oreochromis niloticus* (Linnaeus, 1758) were collected in Silvana Lake. In the Risoleta Neves hydroelectric reservoir, *Oreochromis niloticus*, *Poecilia reticulata* Peters, 1859 and *Hoplosternun litoralle* (Hancock, 1828) were captured in two field studies.

## Discussion

*Plagioscion squamosissimus* is native to the Amazonian region [22], and *P. managuensis* is native to Central America [23]. The piscivorous *Plagioscion* is considered to be a valuable resource for human consumption and recreational fishing [22]. *Parachromis managuensis* is also piscivorous and indigenous to Honduras and Costa Rica, where it reaches a maximum length of 65 cm [23]. The first individual of *P. managuensis* was captured in Brazil in the middle São Francisco River Basin [24]. It is noteworthy that the presence of *P. managuensis* in the Doce River Basin was associated with eutrophic conditions, which are suitable for the ecological success of this invasive species. Considering the great commercial and sport appeal of *P. squamosissimus*, its introduction in the Doce River was most likely due to aquaculture activities. Fingerlings of this species are currently commercially available, as are fingerlings of *Cichla temensis* Humboldt, 1821 and *Oreochromis* spp. [24].

The presence of 35 exotic species in the upper and middle section of the Doce River Basin [19], [25]–[27] (Table 1), like *Cichla monoculus*, *Oreochromis niloticus*, *Poecilia reticulata* and *Hoplosternun litoralle* suggests this basin has favorable environmental conditions for successful fish invasions. This basin is also characterized by a high proportion of species with restricted distribution (48%), a factor that seems to favor the establishment of exotics. The same pattern applies to the Mediterranean basins [3]. In the middle Doce River Basin, the introduction of non-native species has been followed by a steep decline in native fish communities [28]. Surveys conducted in the local communities of the middle Doce River show that 84% of responses indicate an increase in the number of non-native species and 82% perceive a decline in the native species *Astyanax* spp., *Leporinus steindachneri* Eigenmann, 1907 and of the characin predatory *Hoplias malabaricus* (Bloch, 1794) [21]. *Plagioscion squamosissimus* has no close phylogenetic relationships with native predatory sciaenid fishes of this region, which may favor its success. The ecology of the native sciaenid, *Pachyurus adspersus* Steindachner, 1879 is unknown, but it is usually caught with earthworms as bait, suggesting that this species is a bottom feeder. Likewise, *P.managuensis* has no equivalent native cichlid piscivorous species in the Doce River Basin. Alien species phylogenetically close to native ones can be controlled by natural enemies, which reduces the success of their establishment [29]. However, the lack of competitive and anti-predation strategies of native species against exotics favors the establishment of the latter [30].

The first exotic fish species reported for the Doce River Basin were the piscivorous *Pygocentrus nattereri* Kner, 1858 and *Cichla monoculus*, introduced in the 1960s in the Jacaré and Barra lakes, respectively [25]. In the following years, these species dispersed to most of the middle Doce River lakes through channels connecting the lakes and intentional releases by locals. The trend of temporal reduction of species richness in lakes with exotic fishes is documented in studies from the 1990s. The decrease in species richness between 1983 and 1992 was observed in Jacaré, Carioca and Dom Helvécio lakes and attributed to the introduction of *P. nattereri* and *C. monoculus*. In 1992 the decline of small-sized species and native species' juveniles was correlated to the increase in exotic biomass, unlike the conditions seen in Ferrugem, Timboré and Palmeiras lakes, which lack alien fishes [25]. In 2000, studies in Bonita, Gambá and Águas Claras lakes (with non-native fishes) showed lower fish species richness than Gambazinho, Azul and Lagoinha lakes (without non-native fishes). High levels of capture of non-native predator species in aquatic macrophytes, the natural shelter of native species, were also observed in lakes with alien fishes indicating that *P. nattereri*, *C. monoculus* and *Astronotus ocellatus* (Agassiz, 1831) also compete with native fishes for shelter [19]. Changes in lower trophic cascade due to the introduction of non-native piscivorous fishes were observed in the Doce River Basin. The reduction in species richness of zooplankton over 25 years (1980–2005) occurred in the Carioca and Dom Helvécio lakes. In Carioca Lake, all species of cladocerans disappeared from the limnetic zone. In Dom Helvécio Lake several species of calanoids are no longer found [27]. In these lakes the introduction of *P. nattereri* and *C. monoculus* apparently increased the abundance of invertebrate predators such as *Chaoborus*. These organisms replaced the planktivorous fish in the trophic cascade as a result of an “ecological release”, allowing major energy flow in aquatic ecosystems with exotic fish introduction [27].

*Plagioscion squamosissimus* study feeding in Tibagi River, Paranapanema Basin, Paraná State, Brazil showed a carnivorous diet with preference for fish but also including shrimp, Odonata and Ephemeroptera in areas with low fish density. *Plagioscion squamosissimus* was first recorded in the Paranapanema River Basin in 1992. In 2006, this species showed high abundance and distribution in this basin [31]. Larvae-feeding studies of *P. squamosissimus* in the Itaipu hydroelectric reservoir (Brazil-Paraguay) showed high rates of predation of prey with sizes greater than those observed in native species [32]. *Parachromis managuensis* is a highly aggressive piscivore and an alien species for the São Francisco Basin [24]. This feeding behavior also characterizes *C. monoculus* and *P. Nattereri*, indicating that these two new species are real threats to the fish fauna of the Doce River Basin.

The presence of alien species is reducing the abundance of native species in the middle Doce River [19], [20], [25], [26], [33], [34]. A similar situation has been observed for native fishes in Victoria Lake, Africa, after the introduction of *Lates niloticus* (Linnaeus, 1758) [35] and in other biodiversity hotspots invaded by alien fishes [36]. The native fish fauna of the middle Doce River survives only in lakes without alien species, but the relative lack of appeal of native species to local people can jeopardize the integrity of the lake system. Avoiding introductions of exotic species is not a great concern among fishermen, although many locals acknowledge possible damage caused by introduced species such as *Cichla monoculus*, *Oreochromis niloticus*, and *Colossoma macropomum* (Cuvier, 1818), which are exploited commercially and for sport fishing [21]. The lack of popular awareness of the value of native biodiversity has contributed to the spread of exotic fish species and subsequent biodiversity loss in Doce River Basin. Sport fishing and aquaculture activities also favor the introduction of *P. squamosissimus* and *P. managuensis*, which have already impacted other watersheds where they were introduced. Invasions by non-native piscivorous fishes have been historically observed in Doce River Basin and these two exotic species are likely to aggravate biodiversity loss of the Rio Doce Basin in Minas Gerais, Brazil.
